# Indoor solid fuel use and tuberculosis in China: a matched case-control study

**DOI:** 10.1186/1471-2458-11-498

**Published:** 2011-06-25

**Authors:** Xiaohong Kan, Chen-Yuan Chiang, Donald A Enarson, Wenhua Chen, Jianan Yang, Genwang Chen

**Affiliations:** 1 Anhui Provincial Tuberculosis Institute, Jixi Road, Hefei. 230022, Anhui, China; 2 International Union Against Tuberculosis And Lung Disease, boulevard Saint-Michel, 75006, Paris, France; 3 Department of Internal Medicine, Wan Fang Hospital, Taipei Medical University, Hsin-Long Road, Taipei City, 116, Taiwan; 4 Department of Internal Medicine, School of Medicine, College of Medicine, Taipei Medical University, Wu-Hsing Street, Taipei City, 110, Taiwan; 5 Huai Yuan Center for Disease Control, Shengquan Road, Guobai New City, 233400, Huaiyuan County, Anhui, China

**Keywords:** biomass, fossil fuels, indoor air pollution, risk factors, tuberculosis

## Abstract

**Background:**

China ranks second among the 22 high burden countries for tuberculosis. A modeling exercise showed that reduction of indoor air pollution could help advance tuberculosis control in China. However, the association between indoor air pollution and tuberculosis is not yet well established. A case control study was conducted in Anhui, China to investigate whether use of solid fuel is associated with tuberculosis.

**Methods:**

Cases were new sputum smear positive tuberculosis patients. Two controls were selected from the neighborhood of each case matched by age and sex using a pre-determined procedure. A questionnaire containing demographic information, smoking habits and use of solid fuel for cooking or heating was used for interview. Solid fuel (coal and biomass) included coal/lignite, charcoal, wood, straw/shrubs/grass, animal dung, and agricultural crop residue. A household that used solid fuel either for cooking and (/or) heating was classified as exposure to combustion of solid fuel (indoor air pollution). Odds ratios and their corresponding 95% confidence limits for categorical variables were determined by Mantel-Haenszel estimate and multivariate conditional logistic regression.

**Results:**

There were 202 new smear positive tuberculosis cases and 404 neighborhood controls enrolled in this study. The proportion of participants who used solid fuels for cooking was high (73.8% among cases and 72.5% among controls). The majority reported using a griddle stove (85.2% among cases and 86.7% among controls), had smoke removed by a hood or chimney (92.0% among cases and 92.8% among controls), and cooked in a separate room (24.8% among cases and 28.0% among controls) or a separate building (67.8% among cases and 67.6% among controls). Neither using solid fuel for cooking (odds ratio (OR) 1.08, 95% CI 0.62-1.87) nor using solid fuel for heating (OR 1.04, 95% CI 0.54-2.02) was significantly associated with tuberculosis. Determinants significantly associated with tuberculosis were household tuberculosis contact (adjusted OR, 27.23, 95% CI 8.19-90.58) and ever smoking tobacco (adjusted OR 1.64, 96% CI 1.01-2.66).

**Conclusion:**

In a population where the majority had proper ventilation in cooking places, the association between use of solid fuel for cooking or for heating and tuberculosis was not statistically significant.

## Background

The internationally recommended strategy for global tuberculosis control relies heavily on both efficient case finding of tuberculosis and effective anti-tuberculosis treatment rendering these infectious tuberculosis cases non-infectious [1]. According to the estimates of World Health Organization, the target of halving prevalence and mortality of tuberculosis globally by 2015 compared with the level in 1990 is unlikely to be met with current strategies [2]. It has been proposed that greater emphasis be given to primary preventive activities addressing risk factors of tuberculosis and their social determinants [3]. Indoor air pollution has been listed as one of the major risk factors for tuberculosis with an estimated population attributable fraction of 26.2% (95% confidence interval (CI) 12.4 - 61.0) in the 22 countries with the highest estimated burden of tuberculosis.

China ranks second among the 22 high burden countries for tuberculosis [3]. A modeling exercise showed that reduction of indoor air pollution could help advance tuberculosis control in China [4]. However, the association between indoor air pollution and tuberculosis is not yet well established [5] and there is no study on the association of indoor air pollution with tuberculosis conducted in China. To investigate the association between indoor air pollution and tuberculosis, a study designed by The International Union Against Tuberculosis and Lung Disease (The Union) was conducted independently in both China and Benin. Those from Benin have been previously reported [6]. We report results of the study in China.

## Methods

This matched case control study was conducted in Huai Yuan county, Anhui, China. The null hypothesis is that exposure to combustion of solid fuel is not more frequent among those with tuberculosis than those without tuberculosis.

Cases were consecutive new sputum smear positive tuberculosis patients (never previously treated for tuberculosis for as much as one month ) aged 15 years and above presenting to health facilities in Huai Yuan County. Two controls were selected by the following pre-defined procedure. Two households were randomly selected in the community where the cases lived, with interview of a key informant from each of the two houses. The last digit (n) of the index patient's registration number was used for selection of the control household. If n was an even number, the n^th ^and (n+1)^th ^neighboring house to the right of patient's house was selected as the first and the second control, and if they refused to participate in the study, the next (n+2, n+3, etc) neighboring house to the right was selected till two neighboring houses were recruited. If n was an odd number, the neighboring houses to the left of patient's house was selected as controls using the same procedures.

One informant was selected from each neighboring house. Any family member of the same sex and similar age (within the range of ± 15 years old) of the patient was of priority selected as a control. Using the assumptions that prevalence of exposure in controls is at least 20%, odds ratio of importance is at least 2.0, and applying a 95% 2-sided confidence interval and 80% power, the number of participants required to satisfactorily address the hypothesis was 137 cases and 274 controls. Case enrollment was started in September 2008 and continued till 202 tuberculosis patients and 404 controls were recruited to account for the effects of other variables and those in whom information collected was incomplete.

After informed consent was obtained, the research technician administered a questionnaire containing demographic information, smoking habits and use of solid fuel for cooking or heating [7]. Solid fuel (coal and biomass) included coal/lignite, charcoal, wood, straw/shrubs/grass, animal dung, and agricultural crop residue; non-solid fuel included electricity, liquefied petroleum gas, natural gas, biogas, and kerosene. A household that used solid fuel either for cooking and (/or) heating was classified as exposure to combustion of solid fuel (indoor air pollution). Household tuberculosis contact was investigated by asking "is there any family member living in the house who has been diagnosed with tuberculosis in the past 5 years?". Other questions included frequency and amount of use of alcoholic beverages, tobacco smoking behaviour, passive exposure to tobacco smoke, type of stove used for cooking, location of cooking and ventilation of cooking place.

Data were entered using EpiData Entry 3.1. STATA Version 8.0 (STATA Corporation, Houston, Texas) was used for statistical analysis. Odds ratios and their corresponding 95% confidence limits for categorical variables were determined by Mantel-Haenszel estimate. Three approaches were undertaken to address missing values for each independent variable in both univariate and multivariate analysis. First, individuals with missing values were assumed as not-exposed and comparison of exposed vs not-exposed/ unknown was done. Second, individuals with missing values were assumed as exposed and comparison of exposed/unknown vs not-exposed was done. Third, individuals with missing values were excluded and analysis was done among individuals without missing values. Fourth, a category of unknown of each variable was included in multivariate analysis. P value less than 0.05 was considered statistically significant. All possible variables regardless of p value in univariate analysis were entered into a multivariate conditional logistic regression model and a final fitted model was determined by backward elimination methods.

The study was reviewed and approved by The Union Ethics Advisory Group (approval number 01/08).

## Results

Table 1 shows age, sex and other characteristics of the 202 tuberculosis cases and 404 controls. The proportion of participants who used solid fuels for cooking was 73.8% among tuberculosis cases and 72.5% among controls. Among cases, 21 (10.4%) used solid fuels for heating (20 used coal and 1 used straw/shrubs/grass), 8 (4.0%) used non-solid fuels for heating, 169 (83.7%) did not heat their house, and 4 (2.0%) did not provide an answer; among controls, 43 (10.6%) used solid fuels for heating (40 used coal, 1 used charcoal and 2 used straw/shrubs/grass), 15 (3.7%) used non-solid fuels for heating, 341 (84.4%) did not heat their house, and 5 (1.2%) did not provide an answer. The proportion of cases who had any family member having been diagnosed with tuberculosis in the previous 5 years was much higher than that of control (19.8% vs 0.7%). The proportion of ever smokers among tuberculosis cases was also higher than that among controls (52.5% vs 39.1%). Of the 264 ever smokers, 4 (1.5%) started smoking at age < 15 years old, 68(25.8%) at age 15-19 years old, 110(41.7%) at age 20-24 years-old, 77 (29.2%) at age 25 years-old or older, 5(1.9%) did not provide an answer (unknown). Of the 264 ever smokers, 208 (78.8%) smoked currently, 38 (14.4%) had not smoked in the past month, and 18 (6.8%) did not provide an answer (unknown). The proportion of cases who used alcoholic beverages daily was 8.9%, and that among controls was 9.7%. Among participants, there was a mean of 3.6 (range 1-10) persons living in their house, and a mean of 2.1 (range 1-6) rooms per house. The proportion of cases who had 3 or more people per room was 9.9%, and among controls 11.9% (p = 0.617).

**Table 1:** Characteristics of cases and controls

	Case	Control
Total	202 (100%)	404 (100%)
Median age (Mean ± Std Dev) y/o	63 (57.7 ± 18.8)	55(53.2 ± 15.4)
Sex		
Male	147 (72.8%)	290 (71.8%)
Female	55 (27.2%)	114 (28.2%)
Solid fuel for cooking		
Yes	149 (73.8%)	293 (72.5%)
No	52(25.7%)	109(27.0%)
Unknown	1(0.5%)	2(1.0%)
Solid fuel for heating		
Yes	21 (10.4%)	43 (10.6%)
No	177 (87.6%)	356 (88.1%)
Unknown	4 (2.0%)	5(1.2%)
Household contact history*		
Yes	40 (19.8%)	3 (0.7%)
No	143 (70.8%)	371 (91.8%)
Unknown	19 (9.1%)	30 (7.4%)
Ever smoke		
Yes	106 (52.5%)	158 (39.1%)
No	94 (46.5%)	244 (60.4%)
Unknown	2 (1.0%)	2 (0.5%)
Daily use of alcohol beverage		
Yes	18 (8.9%)	39 (9.7%)
No	180 (89.1%)	360 (89.1%)
Unknown	4 (2.0%)	5 (1.2%)
Crowding (3 or more people per room)		
Yes	20 (9.9%)	48 (11.9%)
No	181(89.6%)	352(87.1%)
Unknown	1(0.5%)	4 (1.0%)

* Family member with tuberculosis in past 5 years

Table 2 shows the type of fuels used for cooking. The majority used biomass (coal/lignite/charcoal/wood/straw/shrubs/grass/agricultural crop residue, 73.8% among cases and 72.5% among controls). None of them used kerosene or animal dung. Table 3 shows the type of stove used, location, and ventilation among those who used solid fuel for cooking. The majority used a griddle stove (85.2% among cases and 86.7% among controls); only a very small minority used surrounded fire for cooking. The majority have smoke removed by hood or chimney (92.0% among tuberculosis cases and 92.8% among controls). The majority cooked in either a separate room (24.8% among cases and 28.0% among controls) or a separate building (67.8% among cases and 67.6% among controls); only a very small number of participants cooked in a room used for living / sleeping (4.0% among tuberculosis cases and 1.7% among controls). Further, the majority used a room with windows/doors for cooking; only a very small minority cooked in a closed room (3.4% among cases and 1.4% among controls).

**Table 2:** Type of fuels used by study participants

	Case	Control
Total	202 (100%)	404 (100%)
No food cooked at home	2 (1.0%)	2 (0.5%)
Electricity	8 (4.0%)	11 (2.7%)
Liquefied petroleum gas	38 (18.8%)	87 (21.5%)
Natural gas/Biogas	4 (2.0%)	9 (2.2%)
Kerosene	0	0
Coal / lignite	16 (7.9%)	22 (5.5%)
Charcoal	3 (1.5%)	1(0.3%)
Wood	12 (5.9%)	33 (8.2%)
Straw / shrubs / grass	77 (38.1%)	144 (35.6%)
Animal dung	0	0
Agricultural crop residue	41(20.3%)	93 (23.0%)
Unknown	1(0.5%)	2(0.5%)

**Table 3:** Type of stove used, location, and ventilation among those who used solid fuel for cooking

	Case	Control
Total	149 (100%)	293 (100%)
Type of stove used for cooking		
Open fire	0	0
Surrounded fire	5 (3.4%)	9 (3.1%)
Improved single-pot stove	4 (2.7%)	5 (1.7%)
Improved multiple-pot stove	0	1(0.3%)
Griddle stove	127 (85.2%)	254 (86.7%)
Unknown	13 (8.2%)	24 (7.4%)
Smoke removed by hood or chimney		
Yes	137 (92.0%)	272 (92.8%)
No	11(7.4%)	17 (5.8%)
Unknown	1 (0.7%)	4(1.4%)
Location of cooking		
In a room used for living / sleeping	6 (4.0%)	5 (1.7%)
In a separate room	37(24.8%)	82 (28.0%)
In a separate building	101 (67.8%)	198 (67.6%)
Outdoors	1 (0.7%)	1 (0.3%)
Unknown	4 (2.7%)	7 (2.4%)
Ventilation at location of cooking		
Closed room	5(3.4%)	4(1.4%)
Room with eaves spaces	11(7.4%)	43(14.7%)
Room with open windows / doors	100(67.1%)	169(57.7%)
Room with 3 or fewer walls	3 (2.0%)	7 (2.4%)
Other	4(2.7%)	9(3.1%)
Unknown	26(17.5%)	61(20.8%)

Table 4 shows results of univariate analysis among those without missing information. None of the factors, using solid fuel for cooking (odds ratio (OR) 1.08, 95% CI 0.62-1.87), using solid fuel for heating (OR 1.04, 95% CI 0.54 -2.02), and daily use of alcoholic beverages (OR 0.90, 95% CI 0.49 -1.67) was associated with tuberculosis. Considering that women in general spend more time at home and are more likely to be affected by indoor air pollution, analysis of use of solid fuel for cooking was stratified by sex; the association remained not significant among women (OR 0.39, 95% CI 0.11 - 1.43). Ever smoking and household tuberculosis contact were consistently significantly associated with tuberculosis in univariate analysis in all analytic approaches. The odds ratio for the association between ever smoking and tuberculosis is 2.15 (95% CI 1.42 - 3.25) in analysis restricted to participants without missing information, 2.08 (95% CI 1.38 - 3.14) in analysis comparing exposed vs not-exposed/unknown among all participants, and 2.12 (95% CI 1.41 - 3.18) in analysis comparing exposed/unknown vs not-exposed among all participants; the respective figure for the association between having had family member with tuberculosis in past 5 years and tuberculosis is 26.67 (95% CI 8.25-86.20), 26.67 (95% CI 8.25-86.20), and 18.0 (95% CI 7.15-45.35).

**Table 4:** Mantel-Haenszel estimate of the odds ratio, controlling for matched groups

	Odds Ratio	chi-square	P value	95% CI*
Solid fuel for cooking (n = 597)	1.08	5.23	0.78	0.62 - 1.87
Solid fuel for heating (n = 585)	1.04	0.01	0.90	0.54 -2.02
Male (n = 606)	1.23	0.26	0.61	0.55 - 2.80
Ever smoke (n = 594)	2.15	13.63	<0.01	1.42 - 3.25
Daily use of alcohol beverage (n = 582)	0.90	0.10	0.75	0.49 - 1.67
Household tuberculosis contact^+ ^(n = 546)	26.67	68.94	<0.01	8.25-86.20

+ Family member with tuberculosis in past 5 years

* CI: Confidence Interval

To address potential differential risk of infection between those with and those without family member with tuberculosis in past 5 years, analysis was done in a subset of study population who had no family member with tuberculosis in past 5 years. If any case or control of a triplet had prior family TB contact, the triplet was dropped. This created a subset of study population with 159 cases and 318 controls. In this subset, using solid fuel for cooking (OR 1.2, 95% CI 0.8 -1.8) was not significantly associated with tuberculosis; using solid fuel for heating (OR 1.0, 95% CI 0.8 -1.3) was also not significantly associated with tuberculosis.

Even if the cooking fuels were grouped into biomass fuels and other fuels, using biomass fuel for cooking (OR 0.75, 95% CI 0.44 - 1.27) was not significantly associated with tuberculosis.

The association between use of solid fuel and tuberculosis remained not significant in a multivariate conditional logistic model that includes all possible risk factors regardless of p value in univariate analysis. Table 5 shows that in a multivariate conditional logistic regression model containing "household tuberculosis contact", "ever smoking", and "age" as independent variables, household tuberculosis contact is significantly associated with tuberculosis and the adjusted odds ratio (adjOR, 27.23, 95% CI 8.19-90.58) is not much different from the crude odds ratio in univaritate analysis (26.67, 95% CI 8.25-86.20); the association between ever smoking and tuberculosis remains statistically significant (adjOR 1.64, 96% CI 1.01-2.66) but the strength of the association is reduced by 24% as compared with univariate analysis (OR 2.15, 95% CI 1.42 - 3.25). However, including "household tuberculosis contact" and "ever smoking" in a multivariate model may be problematic because of collinearity; "household tuberculosis contact" and "ever smoking" are significantly correlated (Pearson chi square = 6.959, p = 0.008). In a model that contains "ever smoking" and age but not "household tuberculosis contact" as independent variables, the strength of the association between ever smoking and tuberculosis is stronger (OR 1.81, 95% CI 1.20 - 2.73) as compared with the model containing "household tuberculosis contact". The finding of multivariate analysis is consistent in the approaches that classifying unknown as unexposed, classifying unknown as exposed, and including an unknown category of each variable.(data not shown)

**Table 5:** Multivariate conditional logistic regression

	Odds Ratio	Standard deviation	z	P value	95% CI*
Ever smoke	1.64	0.41	2.00	0.05	1.01-2.66
Household tuberculosis contact^+^	27.23	16.70	5.39	<0.01	8.19-90.58
Age	1.06	0.01	4.54	<0.01	1.03-1.08

+ Family member with tuberculosis in past 5 years

* CI: Confidence Interval

## Discussion

A review of the association of indoor air pollution from household use of solid fuels with health effects in China estimated that 420,000 premature deaths in China were attributable to the use of solid fuels [8]. Observed health effects of indoor air pollution include respiratory illnesses, lung cancer, chronic obstructive pulmonary disease, weakening of the immune system, and reduction in lung function. There is no study on the association between indoor air pollution and tuberculosis in China.

There have been substantial numbers of publications convincingly demonstrating the association between exposure to tobacco smoke and tuberculosis,[9-12] but evidence for the association between indoor air pollution and tuberculosis is scarce [5,10]. To our knowledge, there have been 10 published articles on this subject and the results varied substantially [13-21]. All studies investigated whether exposure to combustion of solid fuels was associated with tuberculosis, without separating the transition from exposure to tuberculous infection, and the transition from infection to tuberculosis disease. Of the 10 studies, 6 were conducted in India and one each in Benin, Malawi, Mexico and Nepal. Gupta et al. reported that those who used wood and cow dung cakes were significantly more likely to have tuberculosis (OR 2.54, 95% CI 1.07 -16.04); however, they did not specify how participants were enrolled and did not control for other variables in the analysis except age [14]. Mishra et al. reported that cooking using biomass fuels is significantly associated with tuberculosis (adjusted OR 2.58, 95% CI 1.98 - 3.37) [16]. Tuberculosis cases were identified by the question "Does anyone listed suffer from tuberculosis". This approach failed to take into account the effect of household tuberculosis contact, which was a significant determinant in the current study in China and studies conducted in Benin [6], Malawi [13], and Nepal [20]. Further, they did not control for smoking and drinking in the analysis. Hazra et al. reported a crude OR of 1.2 of unclean fuel but did not indicate a confidence interval [19]. Kolappan et al conducted a nested case-control study and reported an adjusted odds ratio of 1.7 (95% Cl 1.0 - 2.9) for biomass use and tuberculosis [15]. Pérez-Padilla et al reported a significant association between cooking with biomass stoves and tuberculosis (adjusted OR 2.4, 95%CI 1.04- 5.6) [17]. Three studies reported a significant association between use of biomass for cooking and tuberculosis in univariate analysis but the association was no longer statistically significant in multivariate analysis [6,18,20]. Cramping et al. investigated cooking fire exposure and tuberculosis among females in Malawi and reported a non significant result, but they did not specify type of fuels used [13]. Behera et al conducted a case control study on use of solid fuel and pulmonary TB and reported no association was found between type of fuel used and TB [21].

In all studies on indoor air pollution and tuberculosis, use of solid fuels was applied as a surrogate for exposure to combustion of solid fuels. As Smith et al rightly pointed out, this approach may neglect the fact that type of stoves used, characteristics of cooking place (size and ventilation), removal of smoke by a chimney, cooking method, difference in time-activities pattern and weather may affect the level of exposure [22]. Shetty et al. used "having a separate kitchen" as a proxy measure for socio-economic status and reported that "not having a separate kitchen" is significantly associated with tuberculosis (adjOR 3.26, 95%CI 1.25-8.46) [18]. Cramping et al. specified cooking indoors or outdoors but cooking indoors in both wet and dry season was not associated with tuberculosis (OR 0.6, 95%CI 1.25-8.46) [13]. Pokhrel et al. investigated the location of the kitchen, windows in the kitchen and estimated overall ventilation in the kitchen; and found that cases were more likely to cook in unventilated kitchen (52.8% cases vs 35.6% control, OR 2.02, 95% CI 1.31-3.13) but did not find a statistically significant association between use of a biomass stove and tuberculosis after controlling for other variables (adjOR 1.21, 95% CI 0.48-3.05) [20].

In this study in China, the majority used a griddle stove, had smoke removed by hood or chimney, cooked in a separate room or a separate building; only a very small minority used surrounded fire for cooking, cooked in a room used for living / sleeping or cooked in a closed room. This implies that the level of exposure to combustion of solid fuel in the study population is likely to be low, which could partly explain why there is no significant association between use of solid fuel and tuberculosis. The situation in India reported by Mishra et al. was different: "cooking area in many Indian households tend to be poorly ventilated, and about one-half of all households do not have a separate kitchen [16]. Cooking stoves in most households are simple - often just a pit (a U-shaped construction made from mud), or three pieces of brick". Our findings support the approach of Smith et al. in quantifying health risks of indoor air pollution from household use of solid fuels [22]. They defined household-equivalent solid-fuel exposed population as the product of population using solid fuel and ventilation factors and set the ventilation factor at 0.25 for children and 0.5 for adult in China, but 1 for India, under the consideration that the national improved-stove programme in China has disseminated cooking stoves with chimneys to three-quarters of rural households since 1981, resulting in decreased effective exposure to combustion of solid fuels.

The proportion of participants who ever smoked was relatively high in this study and smoking was associated with tuberculosis. However, smoking was not associated with use of solid fuel and higher levels of smoking were not associated with greater use of biomass fuel (data not shown). There was no evidence that smoking was a confounder on the association between use of solid fuel and tuberculosis.

Pokhrel et al reported that use of kerosene stoves for cooking (adjOR 3.36, 95% CI 1.01-11.22) and kerosene lamps as a main light source in the house (adjOR 9.43, 95% CI 1.45-61.32) were associated with tuberculosis [20]. In our study, there was no participant using kerosene either for cooking or heating. Therefore, we are not able to assess whether use of kerosene for cooking or heating is associated with tuberculosis. Pokhrel and colleagues reported the association with use of biomass for heating (adjOR 3.45, 95% CI 1.44-8.27) as an unexpected finding because their study design focused on cooking-fuel use [20]. We asked specific questions concerning heating practice and type of fuel used for heating but did not find use of solid fuel for heating as a significant risk factor for tuberculosis.

There are several strengths of our study. This is a population-based case control study enrolling consecutive smear positive tuberculosis cases who were registered at Huaiyuan County Tuberculosis Dispensary to ensure that persons with tuberculosis in this population have an equal chance of being selected. We enrolled only smear positive tuberculosis cases to avoid outcome misclassification. Further, we used predetermined procedures in selecting controls to avoid selection bias. Although interviewers were aware of the status of case and control, they did not know what the research question was. We asked participants whether there was any family member having been diagnosed with pneumonia, tuberculosis, bronchitis/emphysema or asthma in the past 5 years to divert the attention away from tuberculosis. We collected details of type of fuels, type of stove, location of cooking and factors related to ventilation to avoid misclassification of exposure. There were limitations of this study. First, neighborhood controls automatically entails matching and may introduce selection bias. If neighborhood controls were more like cases with respect to the use of domestic fuels than controls randomly selected from the source population, we may fail to detect the association between exposure to combustion of solid fuels and tuberculosis because such bias generally is toward the null [23]. Second, exposure to combustion of solid fuels was assessed retrospectively, which may involve recall bias. Third, less than 30% of participants were female, which may limit the power in detecting the association between exposure to combustion of solid fuels and tuberculosis because female are more likely to be affected by such exposure. Fourth, we have investigated whether exposure to combustion of solid fuels is associated with tuberculosis, without separating the transition from exposure to tuberculous infection, and the transition from infection to tuberculosis disease and our conclusions are only valid for the full transition and not its components. Finally, there were a few participants with missing values. However, the number of participants with missing values was small and results were consistent across four analytic approaches used to deal with missing values.

## Conclusion

We were unable to confirm a statistically significant association between use of solid fuel and tuberculosis. Because our study population likely had a low level of exposure to combustion of solid fuels, finding of this study may not be generalizable to other settings. This study needs to be repeated in communities where conditions associated with the use of solid fuels are worse than in our study population to assess whether high level exposure to combustion of solid fuel is associated with tuberculosis.

## Competing interests

The authors declare that they have no competing interests.

## Authors' contributions

DAE proposed the study. DAE and CYC designed the protocol of the study. KX, CW, YJ, CG and CYC discussed and translated the protocol into Chinese. KX, CW, YJ, CG conducted the study and collected data. CYC analyzed the data and prepared the first draft of the manuscript. KX, DAE, CW, YJ, and CG revised the draft manuscript. CYC finalize the manuscript. KX, DAE, CW, YJ, and CG gave final approval of the manuscript. The corresponding author (CYC) has full access to all data in this study and takes complete responsibility for the integrity of the data and the accuracy of the data analysis. All authors read and approved the final manuscript.

## Pre-publication history

The pre-publication history for this paper can be accessed here:

http://www.biomedcentral.com/1471-2458/11/498/prepub
